# Adolescent-Initiated Retrospective Glucose Data Review is Associated With Improved Glycemia in Type 1 Diabetes Mellitus

**DOI:** 10.1155/2024/5218915

**Published:** 2024-10-24

**Authors:** David J. Chenoweth, Benjamin A. Palmer, Andrew W. Norris, Michael J. Tansey, Catherina T. Pinnaro

**Affiliations:** ^1^Stead Family Department of Pediatrics, University of Iowa, 200 Hawkins Drive, Iowa City 52242, Iowa, USA; ^2^Fraternal Order of Eagles Diabetes Research Center, Iowa City 52242, Iowa, USA

## Abstract

**Objectives:** Regular retrospective review of glucose data is an important aspect of type 1 diabetes (T1D) management. Continuous glucose monitors (CGMs) facilitate retrospective review by capturing glucose data and generating standardized reports. However, only a minority of adults with T1D retrospectively review their glucose data, and adolescents are understudied. The objectives of this study were to determine the prevalence of self-reported retrospective glucose data review by adolescents with T1D, determine factors associated with self-reported retrospective glucose data review, and assess whether self-reported retrospective glucose data review was associated with improved glycemia.

**Methods:** We conducted a cross-sectional survey of adolescents aged 12–18 years with T1D in conjunction with review of the associated electronic medical record, which included age, sex, date of diagnosis, clinic hemoglobin A1c (HbA1c), type of insurance, and CGM data. The survey included the Hypoglycemia Fear Survey (HFS) and questions regarding habits and attitudes associated with retrospective review.

**Results:** 112 out of 218 eligible individuals completed the survey (51%). Fifty-three percent of adolescents who completed the survey reported that they had engaged in retrospective glucose data review. Of these, 88% of individuals reported that they reviewed data regularly. Age, sex, race, type of insurance, and CGM use were not associated with retrospective review status. Self-report of retrospective glucose data review was associated with improved glycemia as measured by HbA1c and time in range (TIR) compared to adolescents who indicated they do not review glucose data (*p*=0.006 and *p*=0.04, respectively). There was no difference in HFS scores between reviewers and nonreviewers including the behavioral subscale, worry subscale, and total score.

**Conclusions:** Self-report of retrospective glucose data review was associated with improved glycemia as measured by HbA1c and TIR. Adolescent-initiated glucose data self-review does not appear to be driven by fear of hypoglycemia (FoH).

## 1. Introduction

Intensive insulin therapy has been shown to improve glycemia and reduce long-term complications in persons with type 1 diabetes (T1D) [1]. For adolescents in particular, frequent insulin dose adjustments are required for optimal glycemia given dynamic insulin requirements [1–4]. Continuous glucose monitors (CGMs) facilitate regular retrospective review by generating standardized reports [5]. These reports aid patients in making informed changes to their insulin doses, resulting in improved hemoglobin A1c (HbA1c) and quality of life [6, 7]. However, only a minority of adults with T1D and caregivers of children with T1D routinely download and review CGM data [8].

HbA1c among adolescents with T1D remain well above targets [4, 9], even as the use of diabetes technology becomes more prevalent. Retrospective CGM and glucose data review habits of adolescents are understudied and may represent an important area of education and empowerment to improve glycemia.

This single-center cross-sectional study focused on self-report of retrospective glucose data review by adolescents with T1D. The primary aim was to determine the prevalence of self-reported retrospective glucose data review. The secondary aims were to assess factors associated with self-reported retrospective, independent glucose data review and evaluate whether such review was associated with improved glycemia. Our group previously studied caregiver-initiated retrospective CGM data review and found a significant association with self-reported retrospective CGM data review and hypoglycemia fear scores, but not with glycemia [10]. We hypothesized that, by contrast, retrospective glucose data review by adolescents would not be associated with fear of hypoglycemia (FoH), but would be associated with glycemia as measured by HbA1c and/or time in range (TIR).

## 2. Materials and Methods

We distributed an electronic survey to all adolescents attending University of Iowa Healthcare's pediatric diabetes clinic over a 3-month period while simultaneously obtaining relevant diabetes clinical data from participants' medical records. CGM summary data for the 2 weeks prior to the clinic visit was generated for each participant using his/her device's specific platform where applicable. CGMs included in the study included Dexcom G5 and G6 (85%), Freestyle Libre 1 and 2 (11%), and Medtronic Guardian 3 (4%).

### 2.1. Participants

Adolescents (defined as patients aged 12–18 years) with T1D were recruited from the University of Iowa Stead Family Children's Hospital Pediatric Diabetes clinic from 5/24/2022–8/12/2022. Records for individuals aged 12–18 years were screened using the international classification of diseases, tenth revision (ICD-10) diagnostic code E10, and diagnosis was validated by the research team. Additional inclusion criteria included an understanding of written English. We identified 218 eligible participants.

### 2.2. Procedures

Ethical approval was obtained from the University of Iowa Institutional Review Board. Surveys were distributed and data were managed using Research Electronic Data Capture (REDCap) [11, 12]. REDCap is a secure, web-based software platform designed to support data capture for research by providing an interface for validated data capture, audit trails for tracking data manipulation and export procedures, and automated export to statistical packages.

Participants were offered a tablet to complete the electronic-based survey while in clinic or were asked to provide an email address to which the survey could be sent. Automated email reminders were sent weekly for 2 weeks. Embedded in the survey was a unique ID, enabling linkage to relevant demographic and clinical data.

### 2.3. Measures

The survey collected information regarding attitudes and behaviors associated with retrospective review and insulin dose adjustment. The survey also included the Hypoglycemia Fear Survey (HFS) [13], a validated instrument for quantifying FoH. Higher scores indicate a greater FoH. It has been modified and validated for use in youth with T1D [14]. The complete survey can be viewed in the Supporting Information.

Relevant clinical information was obtained from participants' electronic medical record and linked through a unique ID to their survey responses in REDCap. Information collected included most recent clinic HbA1c, diabetes diagnosis date, and demographic information including sex, race/ethnicity, and type of health insurance. CGM metrics for the prior 2 weeks were also collected if available. These included TIR (glucose: 3.83−9.99 mmol/L), time below range (TBR; considered glucose <3.83 mmol/L), time above range (TAR; considered glucose >9.99 mmol/L), coefficient of variation (the standard deviation of glucose readings over the last 2 weeks divided by the average glucose), and glucose management indicator (average glucose level represented as a percentage according to a formula derived by regressing HbA1c values against mean sensor glucose levels [15]).

### 2.4. Statistical Analysis

Analyses were conducted using R version 4.2.1 (R Core Team (2022). R: A language and environment for statistical computing. R Foundation for Statistical Computing, Vienna, Austria. URL: https://www.R-project.org/). Normality of data was assessed by direct visualization and using the Shapiro–Wilk test. Normally distributed continuous variables were compared using Student's *t*-test. Nonnormally distributed continuous variables were compared using the Mann–Whitney *U*-test. Categorical data was compared using Pearson's *χ*^2^ test.

## 3. Results

### 3.1. Participant Characteristics

Fifty-one percent (112 of 218) of eligible adolescents responded to the survey. Median last clinic HbA1c for adolescents who responded to the survey (responders) was similar than those who did not (nonresponders). The median for both groups was above recommended targets (60 mmol/mol for responders and 62 mmol/mol for nonresponders) [4, 9]. There was no difference in median age, sex, race, insurance type, and CGM use between responders and nonresponders.

Of the 112 adolescents who responded to the survey, 96 completed the entire survey. Fifty-one responders (53%) reported that they had engaged in retrospective review of their blood glucose data with the intent of considering insulin dose changes. These adolescents will subsequently be referred to as reviewers. Of these 51 reviewers, 44 were considered regular reviewers based on their reported frequency of data review (i.e., indicated they review daily, weekly, or monthly). There were no significant differences in demographic information or diabetes-related variables when comparing reviewers with nonreviewers. See Table 1 for detailed comparison of reviewers and nonreviewers.

There was no difference in CGM data availability for reviewers compared to nonreviewers (73% versus 58%, *p*=0.2).

### 3.2. Diabetes Outcomes and Data Review

**Figure 1:**
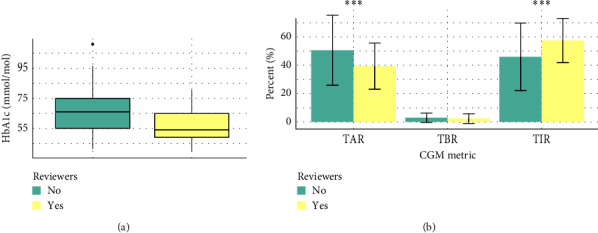
(a) Box and whisker plot demonstrating the significant difference in HbA1c in reviewers and nonreviewers. Boxes represent the 25–75^th^ percentile of HbA1c values. Median is denoted by the black horizontal line. Mann–Whitney *U*-test was performed. (b) Bars demonstrate the mean of each CGM metric; error bars demonstrate the SD. TBR was not normally distributed. Two sample *t*-test was performed on normally distributed data. Mann–Whitney *U*-test was performed on nonnormally distributed data. Asterisks indicate significant difference between reviewers and nonreviewers. CGM, continuous glucose monitor; HbA1c, hemoglobin A1c; SD, standard deviation; TAR, time above range; TBR, time below range; TIR, time in range. *⁣*^*∗∗∗*^indicate significant difference between reviewers and non-reviewers.

HbA1c was significantly lower (52 mmol/mol versus 65 mmol/mol, *p*  < 0.001) and TIR was significantly higher (57.5% versus 45.9%, *p*=0.02) in reviewers compared to nonreviewers (Figure 1a,b). Significance persisted after false discovery rate (FDR) adjustment. The difference in TIR was driven by TAR, and as such, there was no significant difference in TBR.

**Figure 2:**
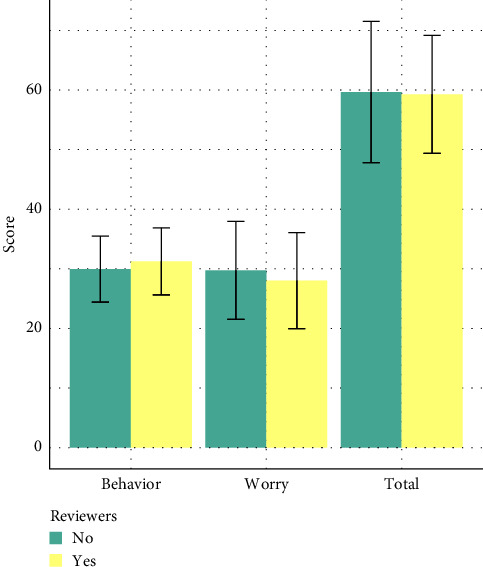
Bar plots demonstrating the mean behavior, worry, and total HFS scores for reviewers and nonreviewers. Error bars demonstrate the SD. Worry subscale was not normally distributed. Two sample *t*-test was performed on normally distributed data. Mann–Whitney *U*-test was performed on nonnormally distributed data. HFS, Hypoglycemia Fear Survey; SD, standard deviation.

There was no difference in the number of episodes of self-reported severe hypoglycemia events between groups (data not shown). There was no difference in HFS scores including behavior subscale, worry subscale, or overall scores between reviewers and nonreviewers (Figure 2).

## 4. Discussion

A minority of adults with T1D and caregivers of children with T1D routinely download and review CGM data [8, 10]. Retrospective CGM and glucose data review behaviors and attitudes are understudied in adolescents with T1D. To our knowledge, we present the first study to describe patterns of self-reported habits of retrospective glucose data review among adolescents with T1D. In contrast to caregiver-initiated review [10], this current study found that self-reported adolescent-initiated retrospective glucose data review was associated with glycemia as measured by HbA1c and TIR.

FoH represents a potential barrier to glycemia in T1D [16]. The HFS has proven a critical tool in exploring the psychological impact of diabetes management on families of children with T1D [17, 18]. Data on glycemia and FoH are mixed, but several studies have demonstrated an association of both higher parental HFS [18–21] and child HFS [22] with higher HbA1c. In our prior study of caregiver-initiated glucose data review, we found a positive association between HFS scores (behavior subscale and total score) and self-reported data review [10]. This suggests that the motivation for caregiver-initiated review may be more related to avoidance of hypoglycemia rather than mitigation of hyperglycemia. In contrast to our prior findings, in the current study there was no difference in HFS scores (or subscale scores) between adolescent reviewers and nonreviewers. Other investigators have demonstrated that parents experience higher levels of FoH than their children [23]. This may suggest that adolescents are less driven by FoH and could potentially explain why adolescent-initiated self-reported review appears to be associated with improved glycemia, because their motivation for review is more focused on correction of hyperglycemia.

Despite the critical role of CGMs in the modern management of T1D, only 20% of adults with T1D and 40% of caregivers with T1D report routinely downloading and reviewing CGM data [8]. Interestingly, the population described in our study demonstrates a much higher rate of self-reported routine retrospective glucose data review; 53% of adolescents surveyed reported performing retrospective glucose data review with intent to make insulin adjustments. This discrepancy is likely multifactorial and may reflect that those who responded to this electronic-based survey are more likely to be comfortable with engagement with electronic-based software including CGM programs, but it is notable that those who responded to our survey had similar characteristics to the nonresponders. Many of the referenced studies are from the prior 5–10 years, since which time CGM use has significantly advanced [24]. No recent studies have systematically evaluated the current rates of retrospective data review, which may be significantly higher in light of new technology and the acceleration of telehealth.

Age, sex, race, type of insurance, and CGM use were not associated with self-reported retrospective review. However, there may be additional factors not accounted for by our study that segregate with self-reported review status and contribute to the described differences in glycemia between reviewers and nonreviewers. Predictors of glycemia trajectory during the transition to adolescence have been described and include executive functioning, family conflict, blood glucose monitoring frequency, and diabetes self-management behaviors [25, 26]. We did not measure these in our study, and it is possible that these variables could impact self-reported review status. Sex has also been shown to impact glycemia during adolescence [25], but there was no difference in sex between reviewers and nonreviewers. Depression and anxiety are more common in adolescents with T1D and could also contribute to the level of engagement with care and CGM review status which can impact glycemia [27].

Further studies would be useful in understanding the differences in review habits between adolescents and adults with T1D. Wong et. al [8] speculates that higher rates of data review among caregivers versus adults with T1D is the product of device downloading practices in pediatric clinics. Pediatric providers routinely download and review device data with families at each clinic visit and encourage patients to download device data and send to the provider between visits. Adult practices typically download device data without the involvement of the patient or rely on hardcopies of device downloads. Our study is not designed to explain this disparity, but does lend support to this hypothesis, as the adolescents review habits were more similar to the caregivers described in the Wong et al. [8] study.

Clinical practice consensus for the care of adolescents with T1D includes intensive training on diabetes technology [28]. However, the literature regarding specific strategies for CGM use and review strategies that are successful in teens is limited to expert opinions [29, 30]. Further studies are needed to detail behaviors associated with improved glycemia among adolescents who perform retrospective review [28, 31, 32]. Identifying these behaviors will be critical to shaping diabetes technology education [33]. Overall, our findings support the conclusion that efforts to educate adolescents on the benefits of CGM data review may lead to improved glycemia.

Notably, this study is not able to comment on the use of specific CGM or insulin pump technologies, as separating the respondents into these categories would have resulted in insufficient statistical power in the reviewer group. Further studies comparing the effects of retrospective review and insulin adjustments could compare the value of retrospective review between users of different technologies. This information could be especially useful among users of hybrid closed-loop automated insulin delivery systems. Other weaknesses of this study include the self-reported nature of the review, and the lack of additional participant characteristics that could confound the association of self-reported review status and glycemia. Future prospective studies, that potentially also could evaluate efforts to educate adolescents on the benefits of CGM data review, could address this.

## 5. Conclusions

In this study, self-reported retrospective glucose data review was associated with improved glycemia as measured by HbA1c and TIR compared to adolescents who do not review CGM data. Unlike our prior study in caregivers, retrospective review does not appear to be driven by FoH in adolescents.

## Figures and Tables

**Table 1:** Demographic and clinical characteristics stratified by review status (i.e., by response to the question “Have you ever looked back at your blood glucose data with intent of making insulin dose changes?”).

Survey question	All	Reviewers	Nonreviewers	*P* value
	*N* = 96	*N* = 51	*N* = 45	
Age	15	14	15	0.71
Median [IQR]	[13, 17]	[13, 16]	[13, 17]	
Sex (% female)	53.1	58.8	46.7	0.32
Race (% white)	91.6	90.0	93.3	0.54
Duration of diabetes (years)	7.6	7.5	7.8	0.45
Median [IQR]	[3.0, 9.7]	[2.7, 9.6]	[3.9, 10.2]	
CGM use (% yes)	93.8	96.1	91.1	0.32
HbA1c (mmol/mol)	60	52	65	**<** **0.001** ^*∗*^
Median [IQR]	[49, 69]	[46, 64]	[53, 75]	
TIR	54	55	42	**0.02** ^*∗*^
Median [IQR]	[41, 67]	[48, 67]	[36, 69]	
Insulin pump use (% yes)	59.4	60.8	57.8	0.77
Automated pump use (% yes)	29.2	33.3	24.4	0.34
Insulin pump trainer taught how to upload pump data	43.8	51.0	35.6	0.16
Provider discussed importance of routine uploading blood glucose data between visits	—	—	—	0.15
Strongly agree	31.2%	39.2%	22.2%	—
Agree	38.5%	35.3%	42.2%	—
Neutral	17.7%	15.7%	20.0%	—
Disagree	7.3%	5.9%	8.9%	—
Strongly disagree	3.1%	0.0%	6.7%	—
Provider discussed importance of family review of uploaded blood glucose data between visits	—	—	—	0.44
Strongly agree	29.2%	35.3%	22.2%	—
Agree	36.5%	33.3%	40.0%	—
Neutral	20.8%	19.6%	22.2%	—
Disagree	8.3%	5.9%	11.1%	—
Strongly disagree	3.1%	2.0%	4.4%	—
Provider discussed importance of family review of uploaded insulin pump data between visits	—	—	—	0.16
Strongly agree	15.6%	25.5%	4.4%	—
Agree	27.1%	25.5%	31.1%	—
Neutral	16.7%	15.7%	17.8%	—
Disagree	7.3%	3.9%	11.1%	—
Strongly disagree	4.2%	3.9%	4.4%	—
NA—never used a pump	27.1%	25.5%	28.9%	—

*Note*: Range was defined as between 3.89 and 9.99 mmol/L. *p* values reported are from two sample *t*-test for normally distributed data, Mann–Whitney *U*-test for nonnormally distributed data, and Pearson *χ*^2^ test for proportions. Significant results are highlighted in bold and indicated by an Asterisk (*⁣*^*∗*^).

Abbreviations: CGM, continuous glucose monitor; HbA1c, hemoglobin A1c; IQR, interquartile range; TIR, time in range.

## Data Availability

The data used to support the findings of this study are available from the corresponding author upon request.
