# Supplementary material for: Adolescent-Initiated Retrospective Glucose Data Review is Associated With Improved Glycemia in Type 1 Diabetes Mellitus
Source: Pediatr Diabetes. 2024 Oct 24;2024:5218915. doi: 10.1155/2024/5218915 (PMC12017010; doi:10.1155/2024/5218915)
Supplement: Supporting Information — The survey in its entirety can be found in the supporting information. [file 5218915.f1.pdf]

T1D Demographics

Thank you for agreeing to participating in our study. Please complete the 2 questions below related to diabetic ketoacidosis (DKA) and severe hypoglycemia (low blood sugar).

In the last 12 months, how many episodes of diabetic ketoacidosis (DKA) requiring hospitalization have you had? __________________________________

Severe hypoglycemia is an event requiring assistance from another person as a result of altered consciousness or confusion to adminster carbohydrate, glucagon, or other. __________________________________

In the last 12 months, how many episodes of severe hypoglycemia have you had?

Fear of Hypoglycemia- Behavior

Below is a list of things that people with diabetes sometimes DO IN ORDER TO AVOID LOW BLOOD SUGAR. Read each item carefully.Click on one of the numbers that best describes YOUR ACTIVITY.1= never, 2= rarely, 3= sometimes, 4= often, 5= very often

		1= never	2= rarely	3= sometimes	4= often	5= very often
1)	Eat large snacks at bedtime	○	○	○	○	○
2)	Try not to be by myself when my sugar is likely to be low	○	○	○	○	○
3)	Allow my blood sugar to be a little high to be on the safe side	○	○	○	○	○
4)	Keep my sugar higher when I will be alone for a while	○	○	○	○	○
5)	Eat something as soon as I feel the first sign of a low blood sugar	○	○	○	○	○
6)	Take less insulin when I think my sugar might get too low	○	○	○	○	○
7)	Keep my blood sugar higher when I am going to be away from my parents	○	○	○	○	○
8)	Carry some kind of of sugar, drink, or food with me	○	○	○	○	○
9)	Try not to do exercise when I think my sugar is low	○	○	○	○	○
10)	Check my sugar often when I am away from home	○	○	○	○	○

Fear of Hypoglycemia- Worry

Below is a list of concerns that people with diabetes sometimes have. Please read each item carefully.Click one of the numbers that best describes how often you worry about each item.1= never, 2= rarely, 3= sometimes, 4= often, 5= very often*Reaction refers to low blood sugar episode (confusion, sweating, seizure, dizziness, poor concentration, passing out)

		1= never	2= rarely	3= sometimes	4= often	5= very often
1)	Not recognizing that my blood sugar is low	○	○	○	○	○
2)	Not having food, fruit, or juice with me when my blood sugar gets low	○	○	○	○	○
3)	Feeling dizzy or passing out in public because of low blood sugar	○	○	○	○	○
4)	Having a reaction while asleep	○	○	○	○	○
5)	Embarrassing myself because of a low blood sugar	○	○	○	○	○
6)	Having a reaction while I am by myself	○	○	○	○	○
7)	Appearing to be 'stupid' or clumsy in front of other people	○	○	○	○	○
8)	Losing control because of low blood sugar	○	○	○	○	○
9)	No one being around to help me during a reaction	○	○	○	○	○
10)	Making a mistake or having an accident at school because of a low sugar	○	○	○	○	○
11)	Getting in trouble at school because of something that happens when my sugar is low	○	○	○	○	○
12)	Having seizures	○	○	○	○	○
13)	Getting long-term complications from frequent low blood sugars	○	○	○	○	○
14)	Feeling dizzy or woozy when my sugar is low	○	○	○	○	○
15)	Having a reaction	○	○	○	○	○

Diabetes Data Review

Please complete these questions which relate to your diabetes devices and how you use them.

Diabetes data and technology: The following questions relate to the use of diabetes technology (such as continuous glucose monitors and insulin pumps).

Have you ever used a continuous glucose monitor (i.e Dexcom) or flash glucose monitor (i.e. Freestyle Libre or Libre2)?	◯ Yes<br>◯ No
Are you currently using a continuous glucose monitor or flash glucose monitor (i.e., Freestyle Libre or Libre 2)?	◯ Yes<br>◯ No
Please check all of the following devices that you have used IN THE PAST 12 months.	☐ Dexcom G4/5/6<br>☐ Freestyle Libre/2<br>☐ Medtronic Enlite<br>☐ Medtronic Guardian<br>☐ Other continuous glucose monitor<br>☐ Medtronic 630G insulin pump<br>☐ Medtronic 670G insulin pump<br>☐ Other Medtronic insulin pump<br>☐ Omnipod system<br>☐ Omnipod DASH<br>☐ Omnipod 5<br>☐ Tslim X2<br>☐ Other insulin pump<br>☐ InPen smart insulin pen<br>☐ Blood glucose meter
Please select from the following list reason(s) why you are not using a continuous glucose monitor (CGM)? Please check all that apply.	☐ Cost (too expensive)<br>☐ Technical issues (issues with calibration, etc)<br>☐ Alarm fatigue (i.e., alarms go off a lot)<br>☐ Discomfort/pain of the device<br>☐ Lack of perceived benefit in my diabetes care<br>☐ Inaccuracy or unreliability of data<br>☐ Time and inconvenience associated with uploading data for review<br>☐ Ability of my caregivers to see blood sugar data in real time<br>☐ Ability of my caregivers to review blood sugar trends<br>☐ Effect on body image<br>☐ Skin/adhesion issues (rashes, irritation, device won't stay on, etc).<br>☐ Interference with my daily routine/activities<br>☐ Other
Please describe other reason(s) you are not using a continuous glucose monitor.	______________________________
Did the insulin pump trainer teach you how to upload the insulin pump data?	◯ Yes<br>◯ No

If you have used a Dexcom continuous glucose monitor in the 12 months, do you?<br><br>Please check all that apply.	☐ Have an adult caregiver(s) who follow your continuous glucose data (ie they can see your glucose on your phone)<br>☐ Review Dexcom Clarity reports<br>☐ Receive Dexcom Clarity data summary text messages<br>☐ Share data with our clinic remotely
If you review Dexcom clarity reports, how often do you review them?	◯ daily<br>◯ every few days<br>◯ weekly<br>◯ monthly<br>◯ less than monthly
If you receive Dexcom Clarity data summary text messages, how often does receiving a text message prompt you to review a Dexcom Clarity report?	◯ never<br>◯ weekly<br>◯ monthly<br>◯ every few months
If you receive Dexcom Clarity data summary text messages, how often does receiving a text message prompt you to contact your diabetes team?	◯ never<br>◯ weekly<br>◯ monthly<br>◯ every few months
How often do you make insulin dose adjustments after reviewing a Dexcom Clarity report?	◯ Never<br>◯ Every few days<br>◯ Weekly<br>◯ Every few weeks<br>◯ Monthly<br>◯ Every few months
If you are sharing Dexcom data with our clinic, do you have to upload the receiver by plugging it into your computer or does your phone/dexcom app share the data directly with us?	◯ I have to plug in the receiver to my computer to share data<br>◯ My phone/dexcom app shares the data directly
If you have used a Libre or Libre 2 flash glucose monitor in the 12 months, do you?<br><br>Please check all that apply.	☐ Have an adult caregiver(s) who follow your glucose data (ie they can see your glucose on your phone with LibreLinkUpp App)<br>☐ Review Freestyle Libre data summary data on LibreView<br>☐ Share data with our clinic remotely
If you review LibreView reports, how often do you review them?	◯ daily<br>◯ every few days<br>◯ weekly<br>◯ monthly<br>◯ less than monthly
How often do you make insulin dose adjustments after reviewing a Libreview report?	◯ Never<br>◯ Every few days<br>◯ Weekly<br>◯ Every few weeks<br>◯ Monthly<br>◯ Every few months
If you are sharing Libre data with our clinic, do you have to upload the Reader by plugging it into your computer or does your phone/Libre app share the data directly with us?	◯ I have to plug in the Reader to my computer to share data<br>◯ My phone app shares the data directly

Have you ever used a Medtronic insulin pump in automode (i.e. 670G, 770G)?	◯ Yes<br>◯ No
If you have ever used a Medtronic 670G in automode, are you still using it in automode?	◯ Yes<br>◯ No
Have you ever used a Tandem insulin pump with Control IQ (i.e., auto-mode)?	◯ Yes<br>◯ No
Are you still using Control IQ?	◯ Yes<br>◯ No
Have you ever used Omnipod 5 in auto-mode?	◯ Yes<br>◯ No
Are you still using Omnipod 5 in auto-mode?	◯ Yes<br>◯ No
Have you ever used a do-it-yourself artificial pancreas system (DIYAPS), such as Loop, OpenAPS, Android APS, or other?	◯ Yes<br>◯ No
Are you still using a DIYAPS?	◯ Yes<br>◯ No
Have you ever looked back at your blood glucose data from previous day(s) with the intent of making insulin dose changes?	◯ Yes<br>◯ No
If you have looked back at your blood glucose data from previous day(s) with the intent of making insulin dose changes, how often do you review the previous days(s) blood glucose data?	◯ daily<br>◯ every few days<br>◯ weekly<br>◯ monthly<br>◯ other
How do you determine when it is needed?	__________________________
What program or application do you use to review previous day(s) blood sugar data? Please check all that apply.	☐ Dexcom Clarity<br>☐ Tidepool<br>☐ Libreview<br>☐ Carelink (Medtronic)<br>☐ T:Connect (Tandem)<br>☐ Other
What other program(s) or app(s) do you use to review data?	__________________________
If you have looked back at the automated insulin delivery from previous day(s) with the intent of making insulin dose/setting changes, how often do you review the previous day(s) insulin delivery data?	◯ Daily<br>◯ Every few days<br>◯ Weekly<br>◯ Monthly<br>◯ Other<br>◯ Not applicable - I have never used an insulin pump with automation

How do you determine when it is needed?

____

What type of insulin dose adjustments do you make based on review of previous day(s) blood glucose data? Please check all that apply.

- ☐ Increasing long acting insulin dose due to frequent morning high blood sugars
- ☐ Decreasing long acting insulin dose due to frequent morning low blood sugars
- ☐ Increasing pump basal rates due to patterns of high blood sugars
- ☐ Decreasing pump basal rates due to patterns of low blood sugars
- ☐ Increasing meal-time insulin doses to to frequent high blood sugars after meals
- ☐ Decreasing meal-time insulin doses due to frequent low blood sugars after meals
- ☐ Setting temporary basal rates due to high blood sugars
- ☐ Setting temporary basal rates due to low blood sugars
- ☐ Giving extra insulin due to a high blood sugar
- ☐ Decreasing the upcoming meal insulin dose due to expected activity level
- ☐ Manually suspending basal insulin delivery due to low blood sugars

My diabetes care provider has discussed the importance of having a home routine of uploading blood glucose data between visits.

- ◯ strongly agree
- ◯ agree
- ◯ neither agree nor disagree
- ◯ disagree
- ◯ strongly disagree

My diabetes care provider has discussed the importance of family review of blood glucose data uploaded at home between visits.

- ◯ strongly agree
- ◯ agree
- ◯ neither agree nor disagree
- ◯ disagree
- ◯ strongly disagree

My diabetes care provider has discussed the importance of family review of insulin pump data uploaded at home between visits.

- ◯ strongly agree
- ◯ agree
- ◯ neither agree nor disagree
- ◯ disagree
- ◯ strongly disagree
- ◯ Not applicable - I have never used an insulin pump

What diabetes-related tasks do you complete independently? Please check all that apply.

- ☐ Determine appropriate meal insulin dose based on carbohydrates and current blood glucose
- ☐ Give insulin via injection for a meal
- ☐ Determine appropriate correction dose for insulin based on current blood sugar
- ☐ Recognize need for change of meal insulin dose due to review of blood sugar patterns
- ☐ Recognize need for change of basal insulin dose (i.e., long acting insulin) due to review of blood sugar patterns
- ☐ Recognize need for change of correction insulin dose due to review of blood sugar patterns

What diabetes-related tasks do you complete independently? Please check all that apply.	☐ Determine appropriate meal insulin dose based on carbohydrates and current blood glucose<br>☐ Give insulin via pump for a meal<br>☐ Determine appropriate correction dose for insulin based on current blood sugar<br>☐ Recognize need for change of meal insulin dose due to review of blood sugar patterns<br>☐ Recognize need for change of basal rate(s) due to review of blood sugar patterns<br>☐ Recognize need for change of correction factor (insulin sensitivity factor) due to review of blood sugar patterns
